# Draft Genome Sequence of *Herbaspirillum huttiense* subsp. *putei* IAM 15032, a Strain Isolated from Well Water

**DOI:** 10.1128/genomeA.00252-12

**Published:** 2013-02-21

**Authors:** Vanely de Souza, Vitor C. Piro, Helisson Faoro, Michelle Z. Tadra-Sfeir, Vanessa K. Chicora, Dieval Guizelini, Vinicius Weiss, Ricardo A. Vialle, Rose A. Monteiro, Maria Berenice R. Steffens, Jeroniza N. Marchaukoski, Fabio O. Pedrosa, Leonardo M. Cruz, Leda S. Chubatsu, Roberto T. Raittz

**Affiliations:** a Laboratory of Bioinformatics, Professional and Technological Education Sector, Federal University of Paraná, Curitiba, PR, Brazil; b Department of Biochemistry and Molecular Biology, Federal University of Paraná, Curitiba, PR, Brazil

## Abstract

Here we report the one-scaffold draft genome of *Herbaspirillum huttiense* subsp. *putei* strain 7-2^T^ (IAM 15032), which was isolated from well water.

## GENOME ANNOUNCEMENT

*Herbaspirillum huttiense* subsp. *putei* strain 7-2^T^ (IAM 15032) is a betaproteobacterium isolated from well water in Osaka, Japan. It was first described as a new species, *Herbaspirillum putei*, closely related to *H. huttiense* (1). Later, 16S rRNA gene resequencing and DNA-DNA hybridization analysis led to the reclassification of *H. putei* as a subspecies of *H. huttiense*, and it is now named *H. huttiense* subsp. *putei* (2).

The genome sequence of *H. huttiense* subsp. *putei* was determined using mate-paired libraries on a SOLiD4 sequencer (Life Technologies), producing a total of 102,768,904 paired reads of 50 bp. These libraries were used for *de novo* genome assembly using Velvet v.1.2.03 (3). Gap closure was achieved by using in-house scripts.

The *H. huttiense* subsp. *putei* draft genome was assembled in one scaffold containing 32 contigs. The estimated genome size is 5.7 Mb with a 62.2% G+C content. Previously estimated genome size was 3.9 Mb with a 62.1% G+C content (2). Automatic annotation using RAST (4) revealed 5,317 open reading frames (ORFs) covering 86% of the chromosome, 49 tRNA genes, and 2 16S-23S-5S rRNA operons.

Our analysis indicated the absence of *nif* genes, confirming that this species is not a nitrogen fixer. On the other hand, genes involved in nitrate/nitrite metabolism were observed. *H. huttiense* subsp. *putei* is able to grow using nitrate as the sole nitrogen source (data not shown), and analyses indicated genes with high similarity to *H. seropedicae nasA, nirD, nirB, narK*, and *nasFED*, whereas genes *narG, narH, narI, narU*, and *narXL* are not present, reinforcing that this bacterium is capable of reducing nitrate as a nitrogen source (assimilative metabolism) and suggesting that it cannot use nitrate as an electron acceptor in anaerobic respiration (dissimilative metabolism).

The *H. huttiense* subsp. *putei* genome has genes coding for all the enzymes required for the Embden-Meyerhof-Parnas pathway. Genes coding for the Entner-Doudoroff, the pentose phosphate, and the tricarboxylic acid cycle (TCA) pathways were also observed. Although *H. seropedicae* and *H. lusitanum* show two potential pathways for trehalose biosynthesis (5, 6), *H. huttiense* subsp. *putei* seems to have only the pathway involving *otsA* and *otsB*, which may be related to the differences among the environments from which these species were isolated.

Studies on plant interaction of *H. huttiense* subsp. *putei* are not available; however, a gene coding for 1-aminocyclopropane-1-carboxylate (ACC) deaminase was observed, which may suggest contributions to plant development under stress conditions (5). Although secretion systems type I, II, and V were observed, the type III secretion system, which was suggested to be involved in plant-bacterium interaction, was not observed in *H. huttiense* subsp. *putei*. An interesting feature was the presence of a gene cluster for cellulose biosynthesis (*wss*) and degradation that was reported only in *H. rubrisubalbicans* M1 and seems to be involved in enhanced colonization of maize (7).

### Nucleotide sequence accession number.

This Whole Genome Shotgun project has been deposited at DDBJ/EMBL/GenBank under the accession number ANJR00000000. The version described in this paper is the first version, ANJR01000000.
